# XPO1-Mediated EIF1AX Cytoplasmic Relocation Promotes Tumor Migration and Invasion in Endometrial Carcinoma

**DOI:** 10.1155/2022/1361135

**Published:** 2022-12-22

**Authors:** Yuhong Ye, Chengyu Lv, Jiandong Sun, Zihang Lin, Yue Liu, Yuxiu Huang, Yupeng Chen, Hua Li, Xiuli Lian, Xia Jiang, Sheng Zhang, Shie Wang

**Affiliations:** ^1^Department of Histology and Embryology, School of Basic Medical Sciences, Fujian Medical University, Fuzhou 350122, China; ^2^Department of Pathology, The First Affiliated Hospital of Fujian Medical University, Fuzhou 350005, China; ^3^Key Laboratory of Stem Cell Engineering and Regenerative Medicine of Fujian Province University, Fujian Medical University, Fuzhou 350122, China; ^4^Department of Obstetrics and Gynecology, Fujian Maternity and Child Health Hospital, Affiliated Hospital of Fujian Medical University, Fuzhou 350001, China; ^5^Department of Obstetrics and Gynecology, The First Affiliated Hospital of Fujian Medical University, Fuzhou 350005, China

## Abstract

Dysregulation of eukaryotic translation initiation factor 1A, X-linked (*EIF1AX*), has been implicated in the pathogenesis of some cancers. However, the role of *EIF1AX* in endometrial carcinoma (EC) remains unknown. We investigated the *EIF1AX* expression in EC patients and assessed its tumorigenesis-associated function and nucleocytoplasmic transport mechanism *in vitro* and *in vivo*. The results indicated that the cytoplasmic EIF1AX expression showed a gradual increase when going from endometrium normal tissue, simple endometrial hyperplasia, complex endometrial hyperplasia, and endometrial atypical hyperplasia to EC, while vice versa for the nuclear EIF1AX expression. In addition, the cytoplasmic EIF1AX expression was positively correlated with histologic type, high International Federation of Gynecology and Obstetrics (FIGO) grade, advanced FIGO stage, deeper infiltration, high Ki67 index, and shorter recurrence-free survival in EC patients. *In vitro*, short hairpin RNA-mediated *EIF1AX* depletion or SV40NLS-mediated EIF1AX import into the nucleus in multiple human EC cells potently suppressed cell migration and invasion, epithelial-mesenchymal transition, and lung metastasis. Moreover, exportin 1 induced the transport of EIF1AX from the nucleus to the cytoplasm that could be inhibited by leptomycin B treatment or the mutation in the *EIF1AX* location sequence. These results demonstrate that cytoplasmic EIF1AX may play a key role in the incidence and promotion of EC, and thus, targeting EIF1AX or its nucleocytoplasmic transport process may offer an effective new therapeutic approach to EC.

## 1. Introduction

Endometrial carcinoma (EC) is the second most diagnosed gynecologic malignancy and the sixth most diagnosed cancer in women worldwide [1–3]. In China, which is undergoing rapid socioeconomic transitions, the incidence rates of EC have been increasing, and its onset shows a trend toward occurrence in younger women over the past decades [1, 2, 4]. Its risk factors include early menarche, late menopause, nulliparity, obesity, physical inactivity, tamoxifen, polycystic ovary syndrome, positive family history, and genetic alterations [5, 6]. Although treatment strategies have greatly improved, outcomes in EC patients with advanced or recurrent disease are far from satisfactory [5, 7]. Presently, it is more important to clarify the molecular mechanisms underlying the growth, metastasis, and recurrence of EC, which may foster research into potential targets for early diagnosis and gene therapy.

Eukaryotic translation initiation factors (eIFs), which are transported into and out of nuclei through the central channels of nuclear pore complexes (NPC) by nucleocytoplasmic transport receptors, regulate the gene expression. This can lead to the abnormal activation or inhibition of signaling pathways involved in tumor progression metastasis and drug resistance, suggesting eIFs as therapeutic target for various types of cancers [8–10]. Many initiation factors, especially eIF2, eIF3, and eIF4, have been implicated in the etiology of many human cancers [11–15]. However, little is understood regarding the exact role and mechanism of eukaryotic translation initiation factor 1A, X-linked (EIF1AX) in EC. EIF1AX, encoded on human chromosome X, includes a central domain with an oligonucleotide-/oligosaccharide-binding (OB) fold, a small helical subdomain, and two-charged basic and acidic unstructured N-terminal tails (NTTs) and C-terminal tails (CTTs), respectively [16–18]. It is essential for the initiation of protein synthesis, particularly for recruitment of the ternary complex and for assembling the 43S preinitiation complex (PIC) [19–21]. EIF1AX could also augment Ago-mediated Dicer-independent micro (mi) RNA biogenesis and RNA interference [22]. Additionally, it was reported to be one of the major marker genes of mammalian preimplantation zygote genome activation [23]. To our knowledge, EIF1AX is the only example of a PIC subunit that is recurrently mutated in cancer. *EIF1AX* mutations have been reported in uveal melanomas, thyroid tumors, and ovarian carcinomas and are frequently located in the NTT domain [24–27]. Recently, it was found that *EIF1AX* mutations primarily enhance translation of long 5′UTR mRNAs that mainly encode proteins related to cell proliferation, differentiation, angiogenesis, invasion, and metastasis [28]. The nucleocytoplasmic overexpression of EIF1AX was observed in breast cancer and positively associated with its aggressive behavior and worse prognosis. The *EIF1AX* overexpression might promote the G1/S phase transition through the transcriptional repression of *p21* in a p53-independent manner [29]. Further investigation revealed that eIF1A was exported by importin13 (IPO13) in HeLa cells, and an exportin 1 (XPO1)-dependent pathway might also be important for eIF1A localization [30].

In the present study, the *EIF1AX* expression was examined in EC and its precursor lesions and compared with the clinicopathological parameters of EC to explore the role of *EIF1AX* expression. We also observed the effects of EIF1AX knockdown or nuclear entry on the aggressive phenotypes of EC cells and analyzed the relevant molecular mechanisms of nucleocytoplasmic transport of EIF1AX.

## 2. Materials and Methods

### 2.1. Patients

EC (315 cases), simple endometrial hyperplasia without atypia (SEH, 50 cases), complex endometrial hyperplasia without atypia (CEH, 50 cases), endometrial atypical hyperplasia (AEH, 50 cases), and normal endometrium (50 cases) were obtained from the First Affiliated Hospital of Fujian Medical University between Jan 1, 2006 and Aug 31, 2020. None of the patients had undergone chemotherapy, radiotherapy, or adjuvant treatment prior to surgery. The EC in this study included the common endometrioid type (286 cases) and the more aggressive serous carcinomas (29 cases). Of these, 26 samples of endometrial endometrioid carcinoma (EEC), four samples of endometrial serous carcinoma (ESC), and 30 samples of normal endometrium were snap-frozen and stored at −80°C. Pathological diagnosis and International Federation of Gynecology and Obstetrics (FIGO) stage were based on the World Health Organization Classification of Female Genital Tumors (5^th^ edition). The expression of the estrogen receptor, progesterone receptor, p53, and Ki67 was examined during clinicopathological diagnosis. The key demographic and clinical characteristics of the patients are summarized in Table 1.

### 2.2. Animal

Four weeks old athymic nude mice were purchased from Shanghai SLAC Laboratory Animal Co., Ltd. (China) and housed under conditions with controlled temperature (22°C ± 1°C) and light cycle (12 h L +12 h D) for 5-7 days to adapt to the new environment. Experimental protocols concerning mice handling were under the approval of the Institutional Animal Care and Use Committee (IACUC) of Fujian Medical University.

### 2.3. Cell Culture, Stable Cell Lines, and Drugs

HEC-1A and ECC-1 cells were cultured in MYCOY′S5A medium, while RL95-2 cells were cultured in DMEM/F12 medium. All the media were supplemented with antibiotics and 10% fetal calf serum (Gibco, Waltham, MA, USA). To establish stable cell lines, lentivirus-mediated EIF1AX-shRNA, EIF1AXsm, and EIF1AXsm-SV40NLS constructs designed by Shengzhe Biotechnology were each transfected into EC cells according to the manufacturer's protocols. Infected cells were selected with puromycin treatment used in experiments. Leptomycin B (LMB) was purchased from Beyotime Biotechnology (Beijing, China).

### 2.4. Plasmids

The pcDNA3.1-EIF1AX-shRNA, pcDNA3.1-EIF1AXsm, pcDNA3.1-EIF1AX-SV40NLS, pcDNA3.1-EIF1AXsm-SV40NLS, and pcDNA3.1-EIF1AX-NLSmut were all constructed by Hainan Shengzhe Biological Co., Ltd. (Hainan, China). Plasmids were propagated in E. coli DH5*α* and purified using a MN NucleoBond Xtra kit (Macherey-Nagel, Dueren, Germany). All sequences used are listed in Supplementary Figure 1 (Figure S1). Cells were transfected with Lipofectamine 3000 (Thermo Fisher Scientific, USA) according to the manufacturer's instructions and harvested for further experiments after 48 hours.

### 2.5. RNA Interference

The specific small interfering RNA (siRNA) for IPO13, XPO1, and a negative control siRNA was obtained from GenePharm (Shanghai, China). Cells were transfected with Lipofectamine RNAiMAX (Thermo Fisher Scientific, USA) according to the manufacturer's instructions and harvested for further experiments after 48 hours. The siRNA sequences used are listed in Supplementary Table 1.

### 2.6. Immunohistochemistry (IHC) and Scoring

The tissue microarray (TMA) was constructed using tissues from ECs, precursor lesions, and normal endometrium as described by Zhang et al. [31]. After antigen retrieval was conducted by microwaving for 30 min in 0.01 M citrate buffer (PH 6.0), the sections were incubated with anti-EIF1AX antibodies at 1 : 1,000 dilution (Thermo Fisher Scientific, USA, Cat#HPA002561) for 2 h at room temperature, tested with a Dako Envison kit (Dako, Carpinteria, CA, USA), and visualized with diaminobenzidine.

IHC score was graded as previously reported [32–34] and scored independently by two experienced pathologists. In short, the proportion of positive tumor cells was scored as 0 (≤10% positive cells), 1 (≤50% positive cells), 2 (≤75% positive cells), and 3 (≥76% positive cells). Staining intensity was graded as follows: 1 (weak), 2 (moderate), and 3 (strong). A final score was then calculated by the multiple above two scores: 0–1 (-), 2 (+), 3-4 (++), 6 (+++), and 9 (++++). We classified staining results as positive (+ - ++++), low expression (+ – ++), and high expression (+++ – ++++).

### 2.7. Sanger Sequencing

Coding region sequences of *EIF1AX* were sequenced using PCR-based capillary Sanger sequencing described by Martin et al. [17]. All primer sequences for amplification and sequencing are given in Supplementary Table 2.

### 2.8. Western Blot Analysis

Total protein extraction from endometrial tissue samples and EC cells was prepared as previously reported [35]. Nuclear and cytosolic fractions were isolated using Minute™ Cytoplasmic & Nuclear Extraction Kits (Invent Biotechnologies, Inc., Plymouth, MN, USA). Primary antibodies were used at the following dilutions: monoclonal anti-GAPDH-horseradish peroxidase conjugates at 1 : 10,000 (GNI, Japan, Cat#GNI4310-GH-S), anti-EIF1AX antibodies at 1 : 1,000, anti-Snail antibodies at 1 : 1,000 (Proteintech, USA, Cat#26183-1-AP), anti-E-cadherin antibodies at 1 : 1,000 (Cell Signaling Technologies, USA, Cat#14472), anti-IPO13 antibodies at 1 : 1,000 (Novus Biologicals, Cat#NBP1-31508), and anti-XPO1 antibodies at 1 : 1,000 (Santa Cruz Biotechnology, Cat#sc-74454).

### 2.9. EIF1AX Protein Sequence Analysis by cNLS Mapper

Potential nuclear localization signal (NLS) was determinated using the open source software cNLS Mapper (http://nls-mapper.iab.keio.ac.jp/cgi-bin/NLS_Mapper_form.cgi). The NLS scores are calculated with four NLS profiles (for class 1/2, class 3, class 4, and bipartite NLSs), each of which represents a contribution of every amino acid residue at every position within an NLS class to the entire NLS activity [36]. Briefly, a GUS-GFP reporter protein fused to an NLS with a score of 8, 9, or 10 is exclusively localized to the nucleus, that with a score of 7 or 8 partially localized to the nucleus, that with a score of 3, 4, or 5 localized to both the nucleus and the cytoplasm, and that with a score of 1 or 2 localized to the cytoplasm.

### 2.10. Immunofluorescence Analysis

HEC-1A, ECC-1, and RL95-2 cells were treated as described above. Then, cells (1 × 10^5^) were grown on glass coverslips attached to a 24-well plate, subsequently washed three times with 0.1% PVA-PBA, and fixed in 4% paraformaldehyde at room temperature (RT) for 30 min. Cells were then treated with 0.5% Triton X-100 at RT for 30 min, blocked with 3% BSA at RT for 60 min, and then incubated overnight with diluted primary antibodies at 4°C. Cells were then washed with 0.1% PVA-PBS, incubated with Alexa Fluor 488-labeled donkey anti-rabbit secondary antibodies (1 : 1,000 dilution) for 60 min, washed again, and treated with DAPI (1 : 5,000 dilution) for 30 min. Cells were immediately examined under a Leica TCS SP8 confocal microscope. The samples were imaged at lower magnification with high resolution using a ×20/0.80 numerical aperture (NA) objective lens and 2048 × 2048 image pixels with a resolution of 0.25 *μ*m. Primary antibodies were used at the following dilutions: polyclonal rabbit anti-EIF1AX antibodies at 1 : 100 (Thermo Fisher Scientific, Cat#HPA002561).

Photoshop software was used to separate protein expressed in nucleus and cytoplasm according to DAPI images. ImageJ was used to analyze the gray values of immunostaining images. Briefly, images were normalized by the same parameter to subtract background staining. Then, gray values of the nucleus and cytoplasm of each cell were calculated separately.

### 2.11. Cell Counting Kit-8 (CCK-8) Assay

HEC-1A (2,000 cells/100 *μ*L) and RL95-2 cells (3,000 cells/100 *μ*L) were plated in 96-well plates. Cells were divided into four groups with five duplicate wells for each group: Ctrl group (empty vector plasmid), KD group (EIF1AX-shRNA), KD + Esm group (EIF1AX-shRNA+pcDNA3.1-EIF1AXsm), and KD + NLSsm group (EIF1AX-shRNA+pcDNA3.1-EIF1AXsm-SV40NLS). Cell proliferation activity was detected using a CCK-8 assay (MCE, USA) every 24 h. Optical density was detected at 450 nm using a microplate reader.

### 2.12. Wound-Healing Scratch Assay

5 × 10^6^ target cells were transferred into 6-well plates and incubated at 37°C until 80-90% confluence. A 200 *μ*L sterile plastic tip was used to create a wound line across the surface of the wells, and the suspended cells were removed with PBS. Cells were cultured in reduced serum MYCOY′S5A or DMEM/F12 medium in a humidified 5% CO_2_ incubator at 37°C for 48 h, and then images were taken with a phase-contrast microscope. Each assay was replicated three times.

### 2.13. Transwell Migration and Invasion Assay

The migration and invasion assays were performed using transwell chambers (Millipore, Billerica, MA, USA). For migration assays, the transfected cells were seeded into the upper chamber with serum-free medium (2.5 × 10^4^ cells), and the bottom of the chamber contained the MYCOY′S5A or DMEM/F12 medium with 10% fetal bovine serum. For the invasion assay, the chamber was coated with Matrigel (BD Biosciences, Franklin Lakes, NJ, USA), and the subsequent steps were similar to those used in the migration assay. After the cells migrated or invaded for 24 h, they were fixed and stained with crystal violet. Migrated and invaded EC cells were counted under an inverted light microscope. The number of migrated or invaded cells was quantified by counting the number of cells from 10 random fields at ×100 magnification.

### 2.14. Coimmunoprecipitation Assay

Protein lysates were prepared using RIPA lysis buffer (Beyotime Biotechnology, China) supplemented with protease inhibitors. The whole-cell lysates were incubated with 100 *μ*L of protein A or G magnetic beads (Santa Cruz Biotechnology, USA) prebound with anti-EIF1AX (Thermo Fisher Scientific, Cat#HPA002561), anti-FLAG (Millipore, Cat#F7425), anti-IPO13 (Novus Biologicals, Cat#NBP1-31508), anti-XPO1 (Santa Cruz Biotechnology, Cat#sc-74454), normal rabbit IgG control, or normal mouse IgG control. The immunoprecipitates were analyzed using western blotting after elution in sample buffer at 95°C.

### 2.15. Lung Metastases

1 × 10^6^ HEC-1A cells stably expressing plasmids were injected intravenously into the tail vein of nude mice. After 5 weeks (5 animals per groups), the mice were sacrificed. Lungs were collected, fixed in 10% neutral buffered formalin, and embedded in paraffin followed by serial sectioning. Five sections (100 *μ*m apart) from each lung were stained with hematoxylin and eosin (H&E) and photographed. The area of lung metastases was determined by ImageJ.

### 2.16. Statistical Analysis

Statistical analyses were performed using the SPSS 22.0 software program and GraphPad Prism 7.0 (GraphPad Software, Inc., La Jolla, CA, USA). The relationship between IHC expression and clinicopathologic parameters was analyzed using the *χ*^2^ test. Survival analysis was performed using the Kaplan–Meier method and log-rank test. Multivariate survival analysis was performed using the Cox proportional hazards model. Means between the groups or within the groups were compared with the one-way ANOVA. *P* values of <0.05 were considered to be significant.

## 3. Results

### 3.1. EIF1AX Is Overexpressed in the Cytoplasm of Human EC and Has No Mutations in the EIF1AX Coding Region

**Figure 1:**
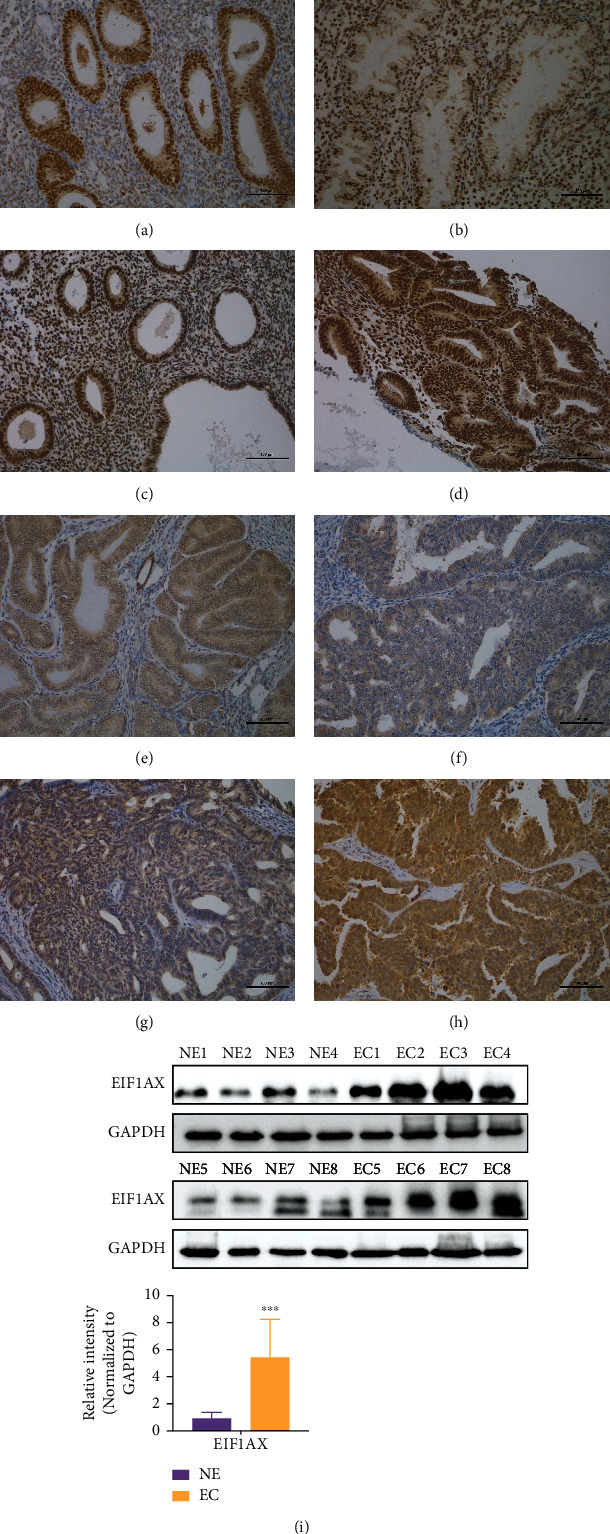
The EIF1AX expression in endometrial carcinoma, precursor lesions, and normal endometrium. EIF1AX protein was positively expressed in normal proliferative phase endometrium ((a) cyt,-; nuc,++++), normal secretory phase endometrium ((b) cyt,-; nuc,+++), simple endometrial hyperplasia ((c) cyt,-; nuc,++++), complex endometrial hyperplasia ((d) cyt,-; nuc,++++), endometrial atypical hyperplasia ((e) cyt,+++; nuc,-), low-grade endometrial endometrioid carcinoma ((f) cyt,+++; nuc,-), high-grade endometrial endometrioid carcinoma ((g) cyt,++++; nuc,-), and serous carcinoma ((h) cyt,++++; nuc,-). Notes: normal endometrium (a, b). Precursor lesions (c–e). Endometrial carcinoma (f–h). Scale bar:100 *μ*m in (a–h). (i) The protein expression of EIF1AX in normal endometrium and endometrial carcinoma tissue. GAPDH was used as a lane loading control. Student's *t*-test: ^∗∗∗^*P* < 0.001. Bars indicate SD. Note: cyt: cytoplasmic expression pattern; nuc: nuclear expression pattern.

Using IHC, we detected the expression of EIF1AX in normal endometrium, precursor lesions, and EC tissues. As shown in Figure 1 and Table 2, compared with normal endometrial cells, the EC cells displayed stronger EIF1AX staining in the cytoplasm (*P* < 0.01), while weaker staining appeared in the nucleus (*P* < 0.01). Additionally, the cytoplasmic EIF1AX expression was higher in EEC than that in normal endometrium, SEH, CEH, or AEH (*P* < 0.01). The nuclear EIF1AX expression was lower in EEC than that in normal endometrium, SEH, CEH, or AEH (*P* < 0.01). Using western blotting, EIF1AX protein levels were found to be higher in EC than in normal tissue, which further confirmed the IHC results. However, Sanger DNA sequence analysis (30 EC patients) did not detect any point mutations, deletions, or insertions in the coding region of *EIF1AX* (Figure S2).

### 3.2. Cytoplasmic EIF1AX Overexpression Is Correlated with Adverse Clinical-Pathological Parameters and Poor Prognosis in Human EC

To determine the clinicopathological significance of the ectopic EIF1AX expression, a comprehensive data set of 315 EC cases was analyzed using a statistical tool. These analyses indicated that EIF1AX protein was mainly located in the cytoplasm of EC cells, with a cytoplasmic positive rate of 82.5%, while the nuclear positive rate was 12.9%. The cytoplasmic EIF1AX expression was positively correlated with histologic type (*P* < 0.01), high FIGO grade (*P* < 0.01), advanced FIGO stage (*P* < 0.01), deeper infiltration (*P* < 0.05), and a high Ki67 index (*P* < 0.01) as shown in Table 1. All cases were followed up periodically for no less than 3 years in total, and cases followed less than 1 year were recorded as lost to follow-up. A total of 143 patients with EC were enrolled in the survival analysis. At a median follow-up of 85 months, 128 patients (89.51%) were alive with no recurrence, four patients (2.80%) were alive but had relapsed, and 11 patients (7.69%) had died. The median survival time was 156 months, and 1-, 3-, and 5-year survival rates were 96.5%, 91.5%, and 86.9%, respectively. The higher the expression of EIF1AX, the worse the overall survival was in EC patients (*P* = 0.000, Figure S3). Univariate analysis using the Cox proportional hazards regression analysis for all parameters showed that high EIF1AX expression, histologic type, histological grade, FIGO stage, invasive depth, and Ki67 index were all prognostic variables in EC patients. However, multivariate analysis confirmed that only histologic type (*P* = 0.003, hazard ratio 1.755–17.022) and Ki67 index (*P* = 0.006, hazard ratio 1.611–17.117) were independent prognostic factors in EC (Table 3).

### 3.3. EIF1AX Knockdown or Relocation to the Nucleus Inhibits EC Cell Proliferation, Migration, and Invasion Capabilities

Given that EIF1AX was mainly overexpressed in the cytoplasm in human EC tissues, we first investigated EIF1AX protein expression and localization in EC cell lines (HEC-1A, ECC-1, and RL95-2). Immunofluorescence staining revealed that the EIF1AX expression in the cytoplasm was stronger than that in the nucleus, especially for HEC-1A and RL95-2 cells; western blot results indicated that the EIF1AX protein expression in HEC-1A and RL95-2 cells was markedly higher than that in ECC-1 cells (Figure S4A, B). Then, we selected HEC-1A and RL95-2 cells as model cell lines for further experiments. To clarify the role of EIF1AX in proliferation of EC cells, we successfully transfected the EIF1AX-NLS plasmid into HEC-1A cells resulting in relocation of the EIF1AX protein to the nucleus, as shown using immunofluorescence and western blotting (Figure S4C, D).

**Figure 2:**
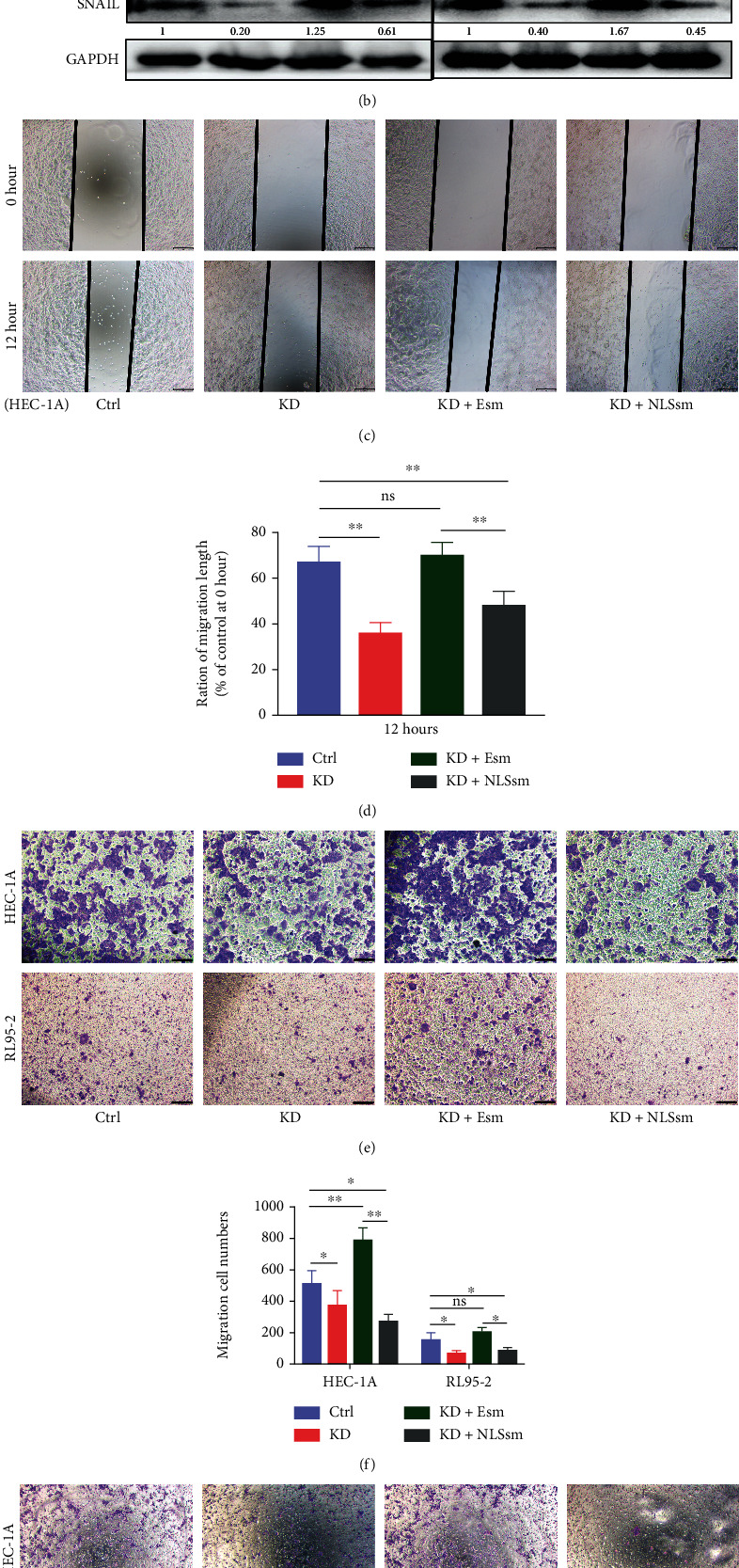
EIF1AX knockdown or translocation into the nucleus inhibits proliferation, migration, and invasion of endometrial carcinoma cells *in vitro*. (a) CCK-8 assay was used to detect cell proliferation activity of HEC-1A (left) and RL95-2 (right) cells at 24 h, 48 h, 72 h, and 96 h. (b) The protein expression of EIF1AX, E-cadherin, vimentin, beta-catenin, and Snail following shRNA transfection. GAPDH was used as a lane loading control. (c, d) HEC-1A cells were transfected with shRNA, and the motility of the cells was evaluated 12 h after transfection using a wound-healing assay. Scale bar: 100 *μ*m. (e–h) HEC-1A and RL95-1 cells described in (c, d) were used in a transwell migration and invasion assay. Scale bar: 100 *μ*m. One-way ANOVA: *n* = 3, ns: *P* > 0.05, ^∗^*P* < 0.05, and ^∗∗^*P* < 0.01. Bars indicate SD. Note: Ctrl group (empty vector plasmid), KD group (EIF1AX-shRNA), KD + Esm group (EIF1AX-shRNA+pcDNA3.1-EIF1AXsm), and KD + NLSsm group (EIF1AX-shRNA+pcDNA3.1-EIF1AXsm-SV40NLS).

We then detected cell proliferation after *EIF1AX* knockdown in EC cells. The results of the CCK-8 assay suggested that cell proliferation activity in the *EIF1AX* knockdown group (KD) was the lowest at 96 h. However, cell proliferation was significantly restored after supplementation of the cytoplasm (KD + Esm group) or nucleus (KD + NLSsm group) with EIF1AX (Figure 2(a)). To further illustrate the role of EIF1AX on migration and invasion of EC cells, we detected the EMT marker gene expression and indicated that Snail and vimentin were decreased, while E-cadherin and beta-catenin were increased after EIF1AX knockdown, implying that EIF1AX may target Snail to promote the epithelial-mesenchymal transition process. Compared with the KD + Esm group, the rescue of EIF1AX in the nucleus did not change the expression of Snail, E-cadherin, vimentin, and beta-catenin more effectively (Figure 2(b)). In addition, we performed wound-healing assays and found that the wound-healing capability of EC cells was reduced after EIF1AX knockdown. Compared with the KD group, the rescue of EIF1AX in the cytoplasm improved its wound-healing capability but not in the KD + NLSsm group (Figures 2(c) and 2(d)). Furthermore, cell migration and invasion assays were performed *in vitro*, and the number of migrating and invading cells was counted. Consistent with the above results, significantly reduced migration and invasion (*P* < 0.05) in the KD and KD + NLSsm groups were observed, implying that downregulation of EIF1AX or relocating it to the nucleus slowed down migration and invasion of EC cells (Figures 2(e)–2(h)). Thus, the different location of EIF1AX between normal tissues and EC may demonstrate that the relocation of EIF1AX to the nucleus is an important factor in EC cell migration and invasion.

### 3.4. EIF1AX Is Transported to the Cytoplasm by the XPO1-Mediated Nuclear Export Pathway in EC Cells

**Figure 3:**
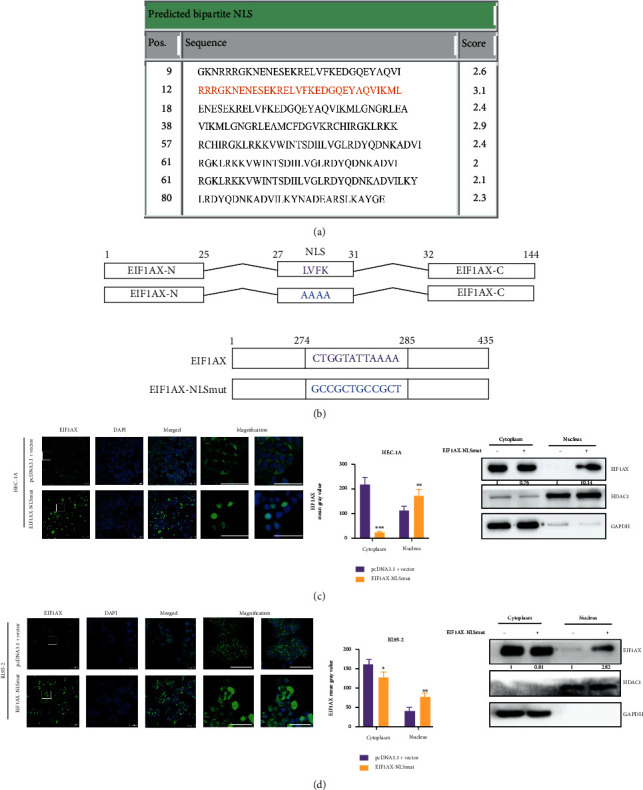
The NLS sequence of *EIF1AX*. (a) The cNLS Mapper Software was used to predict nuclear localization signals (NLSs) of transporters for EIF1AX by calculating NLS scores. (b) Diagram of NLS mutation site in EIF1AX. (c, d) The protein expression of EIF1AX in the nucleus and cytoplasm upon mutation of the NLS sequence in EIF1AX (score 3.1). Scale bar: 100 *μ*m. Scale bar: 50 *μ*m in magnification. Student's *t*-test: *n* = 3, ^∗^*P* < 0.05, and ^∗∗^*P* < 0.01. Bars indicate SD.

In present research, we found that the EIF1AX was located in nucleus in normal tissue. Therefore, the cNLS Mapper was used to predict the NLS sequences of EIF1AX, and the highest scores (score 3.1) of the sequence (RRRGKNENESEKRLVFKEDGQEYAQVIKML) was selected (Figure 3(a)) [36]. Interestingly, the EIF1AX protein was located in the nucleus instead of cytoplasm after the mutant of EIF1AX NLS sequences in HEC-1A and RL95-2 cells (Figures 3(b)–3(d)). In general, small molecules, up to ~20–40 kDa, can passively diffuse across the nuclear pore complexes (NPCs), while other molecules need to be actively transported [37]. Due to the small size (17 kDa), EIF1AX is thought to passively diffuse through the NPCs without NLS sequence; its active export might therefore be required both to deplete EIF1AX from the nucleus and to maintain sufficient cytoplasmic levels [38]. We supposed that the possible overlapping between NLS and NES sequences of EIF1AX resulted in the protein translocation to the nucleus after the mutant of NLS/NES sequence. Michael et al. also found that the NES and NLS activities of M9 are either identical or overlapping as mutants which block M9 NLS activity and also abolish NES activity [39].

**Figure 4:**
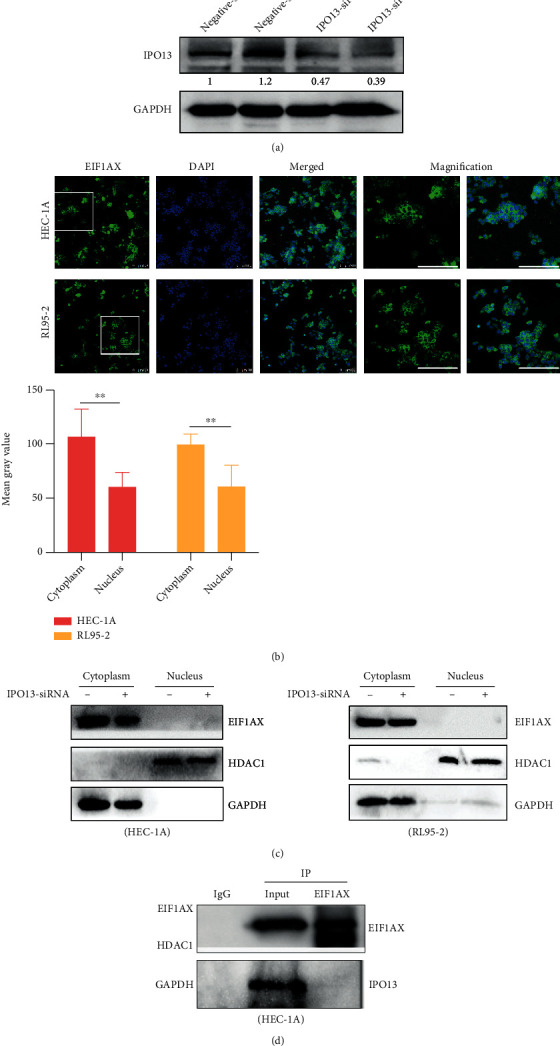
The relationship between IPO13 and EIF1AX. (a) The expression of IPO13 protein after *IPO13* siRNA transfection. (b) Immunofluorescence was used to detect the location of EIF1AX following *IPO13* knockdown. Scale bar: 100 *μ*m. Student's *t*-test: *n* = 3, ^∗∗^*P* < 0.01. Bars indicate SD. (c) The protein expression of EIF1AX in the nucleus and cytoplasm following *IPO13* knockdown. GAPDH was used as the reference gene for the cytoplasm, and HDAC1 was used as the reference gene for the nucleus. (d) Coimmunoprecipitation of EIF1AX with IPO13 in HEC-1A cells.

In order to test the hypothesis above, we further explored the export factors targeted EIF1AX. Previous research points out that IPO13 is a transporter of EIF1AX in HeLa cells [40]; we therefore devised a siRNA targeting IPO13 to suppress the transport process of the EIF1AX protein (Figure 4(a)). However, the location of EIF1AX was not changed after IPO13 knockdown (Figures 4(b) and 4(c)), and the results of the coimmunoprecipitation also showed that EIF1AX did not interact with IPO13 (Figure 4(d)).

**Figure 5:**
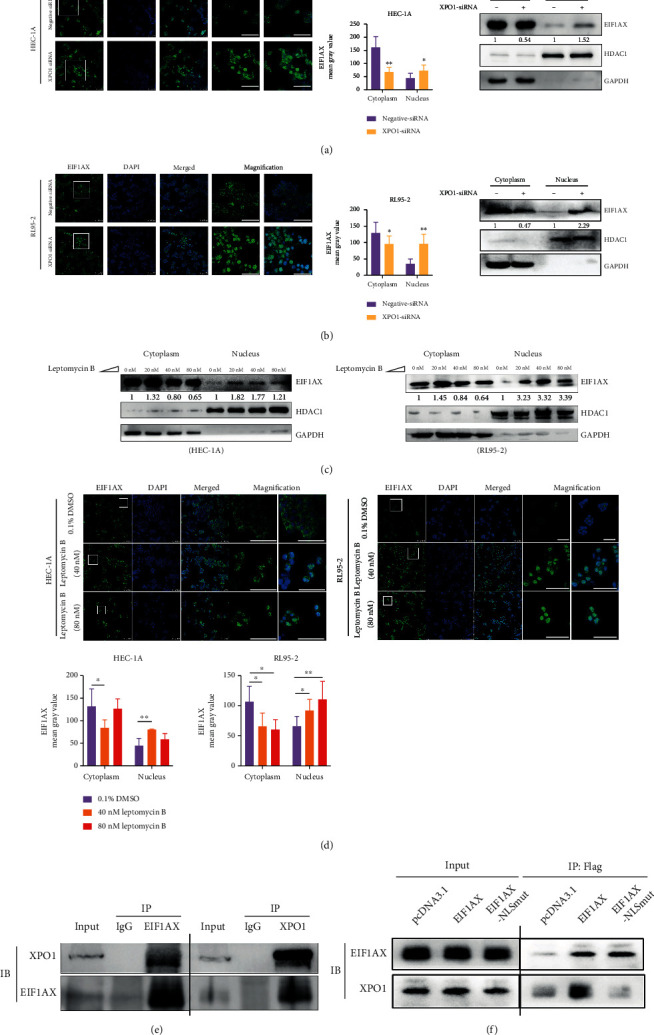
The relationship between XPO1 and EIF1AX. (a, b) The expression of EIF1AX in the nucleus and cytoplasm following *XPO1* knockdown. Scale bar: 100 *μ*m. Scale bar: 50 *μ*m in magnification. Student's *t*-test: *n* = 3, ^∗^*P* < 0.05, and ^∗∗^*P* < 0.01. Bars indicate SD. (c, d) The expression of EIF1AX in the nucleus and cytoplasm following LMB treatment for 1 h. Scale bar: 100 *μ*m. Scale bar: 50 *μ*m in magnification. GAPDH was used as the reference gene for the cytoplasm, and HDAC1 was used as the reference gene for the nucleus. One-way ANOVA: *n* = 3, ^∗^*P* < 0.05, and ^∗∗^*P* < 0.01. Bars indicate SD.(e) Coimmunoprecipitation of XPO1 with EIF1AX in HEC-1A cells. (f) Coimmunoprecipitation of EIF1AX and EIF1AX-NLSmut with XPO1 in HEC-1A cells.

Grunwald et al. predicted that eIF1A export from the nucleus is not unique to IPO13 and might involve other export factors, such as XPO1, and might occur in complex with other proteins [30]. Interestingly, the expression of EIF1AX was increased in the nucleus after XPO1 knockdown (Figure S4E, Figures 5(a) and 5(b)). LMB, a XPO1-cargo formation inhibitor [41], was also used to prove the relationship between EIF1AX and XPO1. EIF1AX was significantly increased (*P* < 0.05) in the nucleus with 40 nM LMB treatment in RL95-2 and HEC-1A cells (Figure 5(c)). Immunofluorescence also demonstrated that EIF1AX was inhibited from translocating to the cytoplasm in the 40 nM LMB groups (Figure 5(d)). However, we also found that the EIF1AX protein expression was reduced in cytoplasm and nucleus after 80 nM leptomycin B treatment in HEC-1A cells but not in RL95-2 cells. Combined with the results of CCK-8, it may be the cytotoxicity of 80 nM leptomycin B to HEC-1A cells (Figure S4F). It suggested that EIF1AX was transported to the cytoplasm by an XPO1-mediated nuclear export pathway in EC cells. In addition, results of the coimmunoprecipitation showed that XPO1 could interact with EIF1AX but not when the NLS (NES) of EIF1AX had been mutated (Figures 5(e) and 5(f)). The findings further implied that the NLS sequences predicted by cNLS Mapper may be the NES sequences or the overlap between them.

### 3.5. EIF1AX Protein Knockdown or Translocation Causes Attenuated Tumor Cell Extravasation *In Vivo*

**Figure 6:**
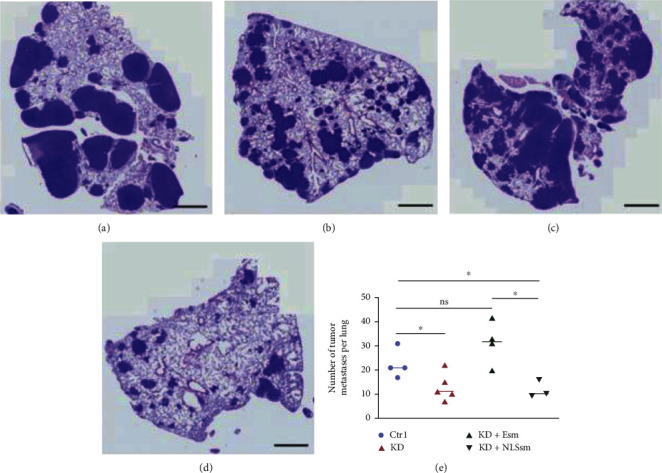
Lung metastases 5 weeks after injection of HEC1A cells in the mice that had received tail vein injection. (a) Ctrl group. (b) KD group. (c) KD + Esm group. (d) KD + NLSsm group. Scale bar: 100 *μ*m. (e) The area of lung metastases were determined by ImageJ. One-way ANOVA: *n* = 3, ns: *P* > 0.05, ^∗^*P* < 0.05. Bars indicate SD. Note: Ctrl group (empty vector plasmid), KD group (EIF1AX-shRNA), KD + Esm group (EIF1AX-shRNA+pcDNA3.1-EIF1AXsm), and KD + NLSsm group (EIF1AX-shRNA+pcDNA3.1-EIF1AXsm-SV40NLS).

The ability of tumor cells expressing wild type or mutant NLS sequence to extravasate into the lung was measured by injecting identical numbers of HEC-1A cells into the tail veins of nude mice. As expected, mice injected with cells expressing EIF1AX shRNA developed shrunken metastatic nodules evidenced both by gross and histological analysis. In addition, compared with the KD + Esm group, mice injected with cells expressing EIF1AX NLS sequence mutant also developed shrunken metastatic nodules (Figure 6). The results also further indicated that EIF1AX translocated to cytoplasm may have an important role in the initiation and progression of EC.

## 4. Discussion

*EIF1AX* was identified as a cancer driver gene in thyroid cancer. *EIF1AX* mutations are present in 11% of poorly differentiated thyroid cancers and anaplastic thyroid cancers and are almost invariably associated with oncogenic RAS mutations [42]. Significant co-occurrence of mutations in *NRAS* and *EIF1AX* was also found in low-grade serous ovarian carcinomas. The coexpression of mutant NRAS and EIF1AX proteins promoted proliferation and clonogenicity survival in LGSC cells [24]. These results imply that *EIF1AX* and *Ras* may drive tumor progression synergistically. In this study, no mutations in the *EIF1AX* coding region were identified. Reasons for this finding may include the small sample number, the limited region assessed, and the sensitivity of the methods used. In agreement with the results in breast and ovarian cancer [24, 29], to our knowledge, we demonstrated for the first time higher ectopic expression levels of EIF1AX protein in EC than in normal tissues and precancerous lesions. The cytoplasmic EIF1AX expression was positively linked to unfavorable clinicopathological characteristics and an adverse prognosis in EC.

The aberrant dysregulation of protein synthesis has been reported to be frequently associated with cancer [43, 44]. EIF1AX plays a key role in scanning and AUG selection and differentially affects translation of distinct mRNAs [20]. EIF1AX also plays an important role in tumor pathogenesis. It has been known that the 5′UTR length is the main feature involved in the translational control by eIF1A in mammalian cells [28]. Cancer-associated eIF1A NTT mutants primarily enhance translation of long 5′UTR mRNAs regulating cell proliferation, differentiation, invasion, metastasis, and angiogenesis [24]. By using an embryonic fibroblast cell model, Urmila et al. showed that cell proliferation significantly declined with the majority of cells arrested in the G1 phase following siRNA-mediated downregulation of *EIF1A* expression levels [28]. In this study, EIF1AX was upregulated in the cytoplasm of EC cells, and EIF1AX knockdown or translocation into the nucleus markedly decreased the ability of EC cells to migrate and invade with E-cadherin overexpression, and Snail hypoexpression at protein levels *in vitro*, and *EIF1AX* knockdown also inhibited proliferation *in vitro*. Our findings were in partial agreement with previous results obtained in thyroid, ovarian, and breast cancer [24, 26, 27, 29]. While the contribution of EIF1AX to tumorigenesis and cancer progression is not clear, EIF1AX has been found to have links to cancer-related signaling pathways including PI3K/AKT/mTOR and Ras/ERK signaling pathways, as well as oncogene c-myc [26, 45–48]. With a deeper understanding of EIF1AX in cancer, EIF1AX may be a good molecular target for gene therapy in the future.

Proteins are known to exhibit diverse biological functions according to their subcellular location; thus, nucleocytoplasmic transport is an essential activity in eukaryotic cells. EIF1AX is localized to both nucleoli and cytoplasm, and its nuclear export process involves specific interactions of transporter IPO13 and EIF1AX localization sequences in HeLa cells [38]. Grunwald et al. subsequently investigated the 3.6-A° crystal structure of IPO13 in complex with RanGTP and with eIF1A and noted that at least a fraction of eIF1A might be exported via a XPO1-dependent pathway [30]. Less consistent with the above, our experiment in EC cells revealed that LMB treatment effectively inhibited XPO1-mediated, but not IPO13-mediated, cytoplasmic export of EIF1AX. The discrepancy among these results may be related to the types of cells used and/or experimental conditions. XPO1 inhibitors are a unique class of drugs and are currently being evaluated in several phase I/II/III clinical studies [49–51]. Recent studies have pointed out that selinexor (an approved inhibitor of XPO1-mediated nuclear export) plus chemotherapy was a safe and tolerated treatment in advanced ovarian and endometrial cancer patients [52, 53]. Our findings may partly provide a theoretical basis for the abovementioned clinical trial results. In addition, we identified the EIF1AX NLS sequence. Indeed, other factors including protein folding conformation, protein posttranscriptional modifications, and protein–protein interactions could influence the recognition and binding between transporters and substrates apart from the amino acid sequence [54, 55].

## 5. Conclusion

In summary, the upregulated expression and nucleocytoplasmic translocation of EIF1AX protein occur in EC. The expression of cytoplasmic EIF1AX was positively correlated with aggressive clinicopathologic features and poor prognosis in EC patients, which might result from the ability of the ectopic EIF1AX expression to facilitate EC cell proliferation, migration, and invasion. The specific location signal sequence of EIF1AX was identified by XPO1 and then transported into the cytoplasm in EC cells. These results indicated that EIF1AX may have an important role in the initiation and progression of EC. Thus, EIF1AX may be employed as a potential target for gene therapy.

## Figures and Tables

**Table 1:** The relationship between cytoplasmic EIF1AX expression and clinicopathological features in endometrial carcinoma.

Clinical parameters		*n*	Cytoplasmic EIF1AX		*P* value
			Negative (-)	Positive (+~++++)	
Histological type	Endometrioid	286	50	236	<0.001
	Serous	29	6	23	
FIGO grade	Grade 1	140	42	98	<0.001
	Grade 2	99	6	93	
	Grade 3	47	2	45	
FIGO stage	I + II	243	53	190	0.001
	III + IV	72	3	69	
Invasive depth	<1/2	176	38	138	0.046
	≥1/2	139	18	121	
Lymphovascular invasion	Negative	222	41	181	0.620
	Positive	93	15	78	
Lymph node metastasis	Negative	178	6	172	0.634
	Positive	27	2	25	
MELF pattern of invasion	Negative	239	38	201	0.112
	Positive	47	12	35	
Ki67 index	Low expression (≤50%)		54	163	<0.001
	High expression (>50%)		2	96	

MELF: microcystic elongated and fragmented.

**Table 2:** The EIF1AX expression in endometrial carcinoma, precursor lesions, and normal endometrium.

Groups	*n*	Cytoplasmic EIF1AX expression						Nuclear EIF1AX expression					
		—	+	++	+++	++++	PR (%)	—	+	++	+++	++++	PR (%)
NE	50	48	0	0	2	0	4.0	2	0	0	2	46	96.0
SEH	50	49	0	0	1	0	2.0	1	0	0	4	45	98.0
CEH	50	40	0	1	9	0	20.0	5	0	0	3	42	90.0
AEH	50	18	5	7	20	0	64.0	8	2	38	2	0	84.0
EEC	286	50	50	100	59	27	82.5^a,b,c,d^	249	21	10	3	3	12.9^a,b,c,d^
EC	315	56	52	104	65	38	82.2^a^	272	22	12	5	4	13.7^a^

^a^ *P* < 0.01, compared with normal endometrium; ^b^*P* < 0.01, compared with SEH; ^c^*P* < 0.01, compared with CEH; ^d^*P* < 0.01, compared with AEH; NE: normal endometrium; SEH: simple endometrial hyperplasia; CEH: complex endometrial hyperplasia; AEH: endometrial atypical hyperplasia; PR: positive rate.

**Table 3:** Univariate and multivariate analyses of the cytoplasmic EIF1AX expression and clinical-pathological factors on survival.

Variables	Univariate		Multivariate	
	HR (95% CI)	*P*	HR (95% CI)	*P*
Cytoplasmic EIF1AX expression (negative vs. positive)	26.313 (0.064-10778.959)	0.287		
Cytoplasmic EIF1AX expression (low vs. high)	3.325 (1.134-9.746)	0.029		
Histological type (endometroid vs. serous)	8.092 (2.790-23.470)	<0.001	5.416 (1.755-17.022)	0.003
Histological grade (low vs. high)	7.722 (2.173-27.442)	0.002		
FIGO stage(I + II vs. III + IV)	6.112 (2.183-17.114)	0.001		
Invasive depth (<1/2 vs. ≥1/2)	5.601 (1.780-17.627)	0.003		
Lymphovascular invasion (no vs. yes)	2.034 (0.721-5.740)	0.180		
Ki67 index (low vs. high)	7.179 (2.283-22.575)	0.001	5.252 (1.611-17.117)	0.006

## Data Availability

All the data is enclosed in the manuscript.

## Abbreviations

EIF1AX: Eukaryotic translation initiation factor 1A, X-linked
EC: Endometrial carcinoma
XPO1: Exportin 1
IPO13: Importin13
LMB: Leptomycin B
FIGO: International Federation of Gynecology and Obstetrics.

## Supplementary Materials

Supplementary 1 Figure S1: schematic diagram showing the insertion sequence of the recombinant plasmid. (A) The site of EIF1AX targeted by shRNA. (B) The homologous sequence of EIF1AX coded by EIF1AXsm plasmid avoids depletion by EIF1AX shRNA. (C) Adding the NLS sequence from SV40 to the C-terminus of EIF1AX. (D) Adding the NLS sequence from SV40 to the C-terminus of homologous EIF1AX. (E) Point mutation site of NLS sequence from EIF1AX. Figure S2: Sanger sequencing results of EIF1AX gene in endometrial carcinoma tissue. Compared with normal tissues, 30 EC patients' tissues did not detect any point mutations, deletions, or insertions in the coding region of *EIF1AX*. Figure S3: the overall survival in endometrial carcinoma patients. Overall survival curves of EC patients with high EIF1AX expression (group 3), low EIF1AX expression (group 2), and the negative control group (group 1). Figure S4: the expression of EIF1AX or EIF1AX-SV40NLS in EC cell lines. (A) Immunofluorescence observed the location of EIF1AX protein in HEC-1A, RL95-2, and ECC-1 cells. Scale bar:100 *μ*m. (B) Western blot detected the expression of EIF1AX in EC cell lines. (C, D) The expression of EIF1AX protein after transfected with EIF1AX-SV40NLS plasmid. Scale bar: 100 *μ*m. Scale bar: 50 *μ*m in magnification. Student's *t*-test: *n* = 3, ^∗^*P* < 0.05, and ^∗∗∗^*P* < 0.001. Bars indicate SD. (E) The expression of XPO1 after *XPO1* knockdown. (F) CCK-8 detected the cell activity of EC cell lines following LMB treatment for 1 h. One-way ANOVA: *n* = 3, ^∗∗∗^*P* < 0.001. Bars indicate SD.

Supplementary 2 Supplementary Table 1: The siRNA sequences targeting IPO13 and XPO1.

Supplementary 3 Supplementary Table 2: primer sequences for amplification and sequencing of EIF1AX-CDS.
