# Supplementary material for: XPO1-Mediated EIF1AX Cytoplasmic Relocation Promotes Tumor Migration and Invasion in Endometrial Carcinoma
Source: Oxid Med Cell Longev. 2022 Dec 22;2022:1361135. doi: 10.1155/2022/1361135 (PMC9800903; doi:10.1155/2022/1361135)
Supplement: Supplementary 1 — Figure S1: schematic diagram showing the insertion sequence of the recombinant plasmid. (A) The site of EIF1AX targeted by shRNA. (B) The homologous sequence of EIF1AX coded by EIF1AXsm plasmid avoids depletion by EIF1AX shRNA. (C) Adding the NLS sequence from SV40 to the C-terminus of EIF1AX. (D) Adding the NLS sequence from SV40 to the C-terminus of homologous EIF1AX. (E) Point mutation site of NLS sequence from EIF1AX. Figure S2: Sanger sequencing results of EIF1AX gene in endometrial carcinoma tissue. Compared with normal tissues, 30 EC patients' tissues did not detect any point mutations, deletions, or insertions in the coding region of EIF1AX. Figure S3: the overall survival in endometrial carcinoma patients. Overall survival curves of EC patients with high EIF1AX expression (group 3), low EIF1AX expression (group 2), and the negative control group (group 1). Figure S4: the expression of EIF1AX or EIF1AX-SV40NLS in EC cell lines. (A) Immunofluorescence observed the location of EIF1AX protein in HEC-1A, RL95-2, and ECC-1 cells. Scale bar:100 μm. (B) Western blot detected the expression of EIF1AX in EC cell lines. (C, D) The expression of EIF1AX protein after transfected with EIF1AX-SV40NLS plasmid. Scale bar: 100 μm. Scale bar: 50 μm in magnification. Student's t-test: n = 3, ∗P < 0.05, and ∗∗∗P < 0.001. Bars indicate SD. (E) The expression of XPO1 after XPO1 knockdown. (F) CCK-8 detected the cell activity of EC cell lines following LMB treatment for 1 h. One-way ANOVA: n = 3, ∗∗∗P < 0.001. Bars indicate SD. [file 1361135.f1.docx]

**Supplemental Figure 1**


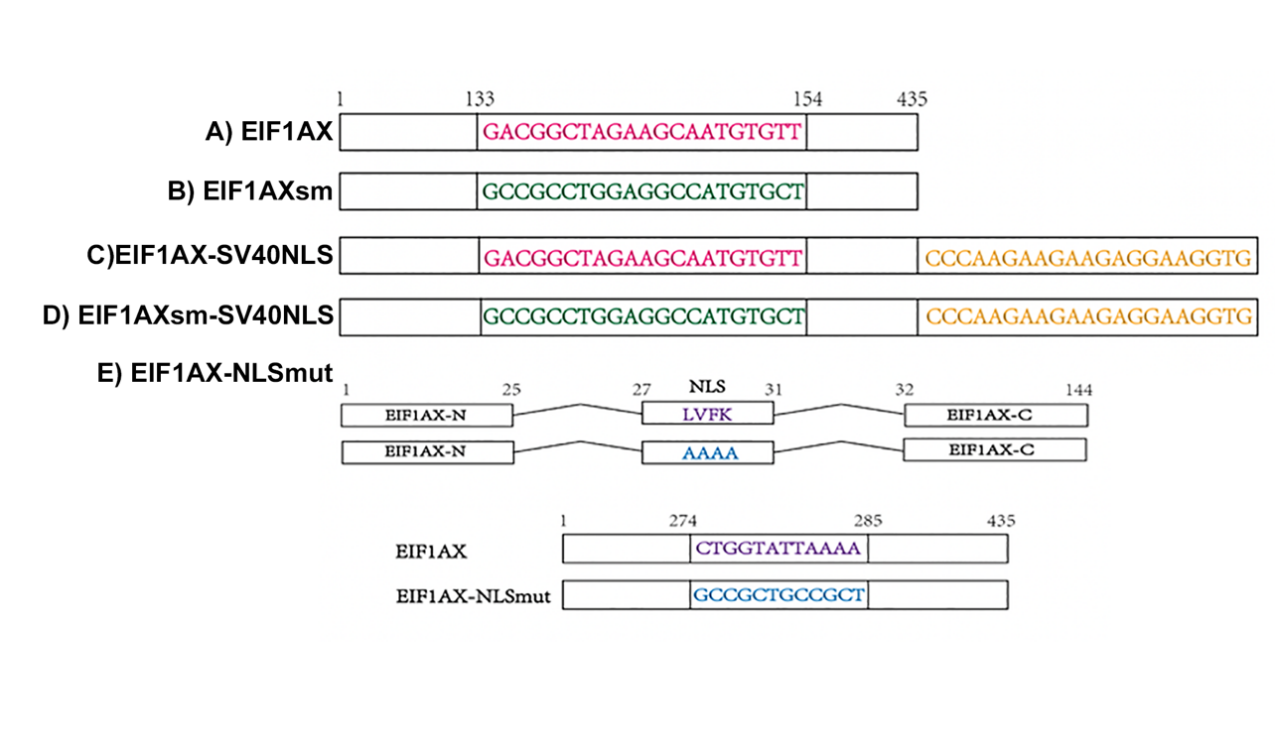


Figure S1. Schematic diagram showing the insertion sequence of the recombinant plasmid. A: The site of EIF1AX targeted by shRNA. B: The homologous sequence of EIF1AX coded by EIF1AXsm plasmid avoids depletion by EIF1AX shRNA. C: Adding the NLS sequence from SV40 to the C-terminus of EIF1AX. D: Adding the NLS sequence from SV40 to the C-terminus of homologous EIF1AX. E: Point mutation site of NLS sequence from EIF1AX.

**Supplemental Figure 2**


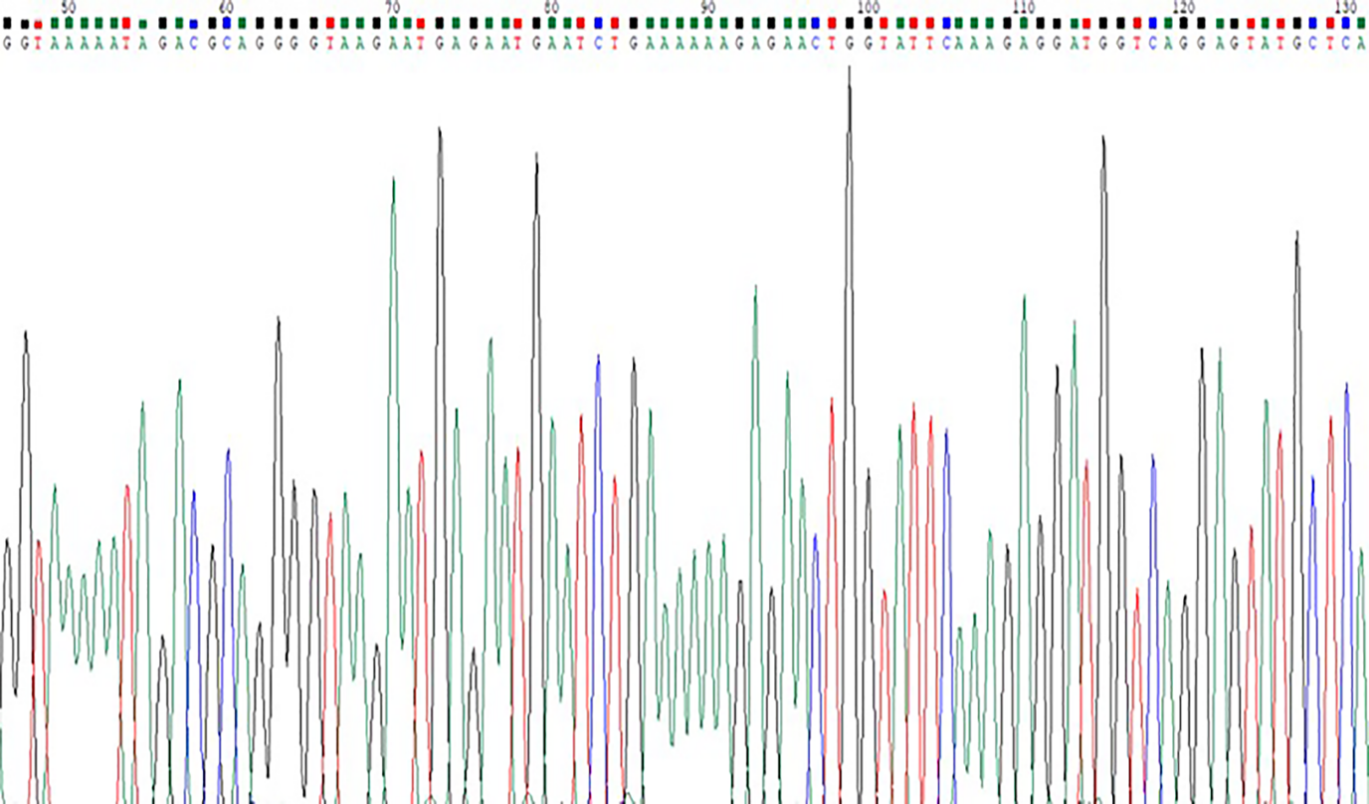


**Figure S2. Sanger sequencing results of EIF1AX gene in endometrial carcinoma tissue.** Compared with normal tissues, 30 EC patients’ tissues did not detect any point mutations, deletions, or insertions in the coding region of *EIF1AX*.

**Supplemental Figure 3**

**
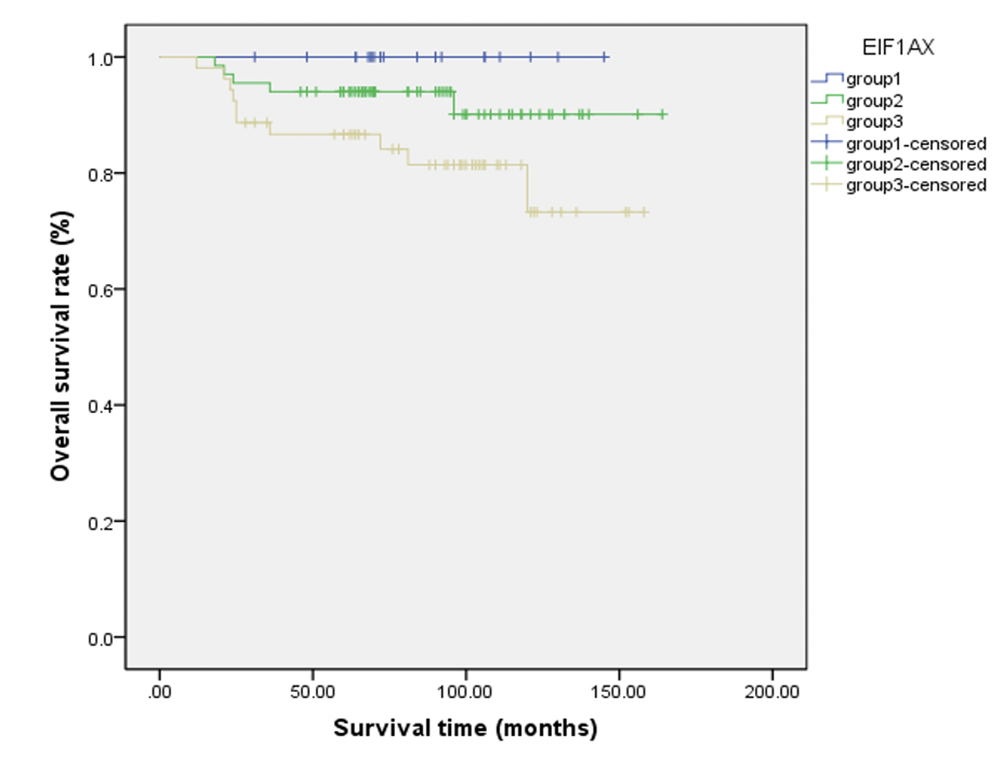
**

**Figure S3. The overall survival in endometrial carcinoma patients.** Overall survival curves of EC patients with high EIF1AX expression (group 3), low EIF1AX expression (group 2), and the negative control group (group 1).

**Supplemental Figure 4**

**
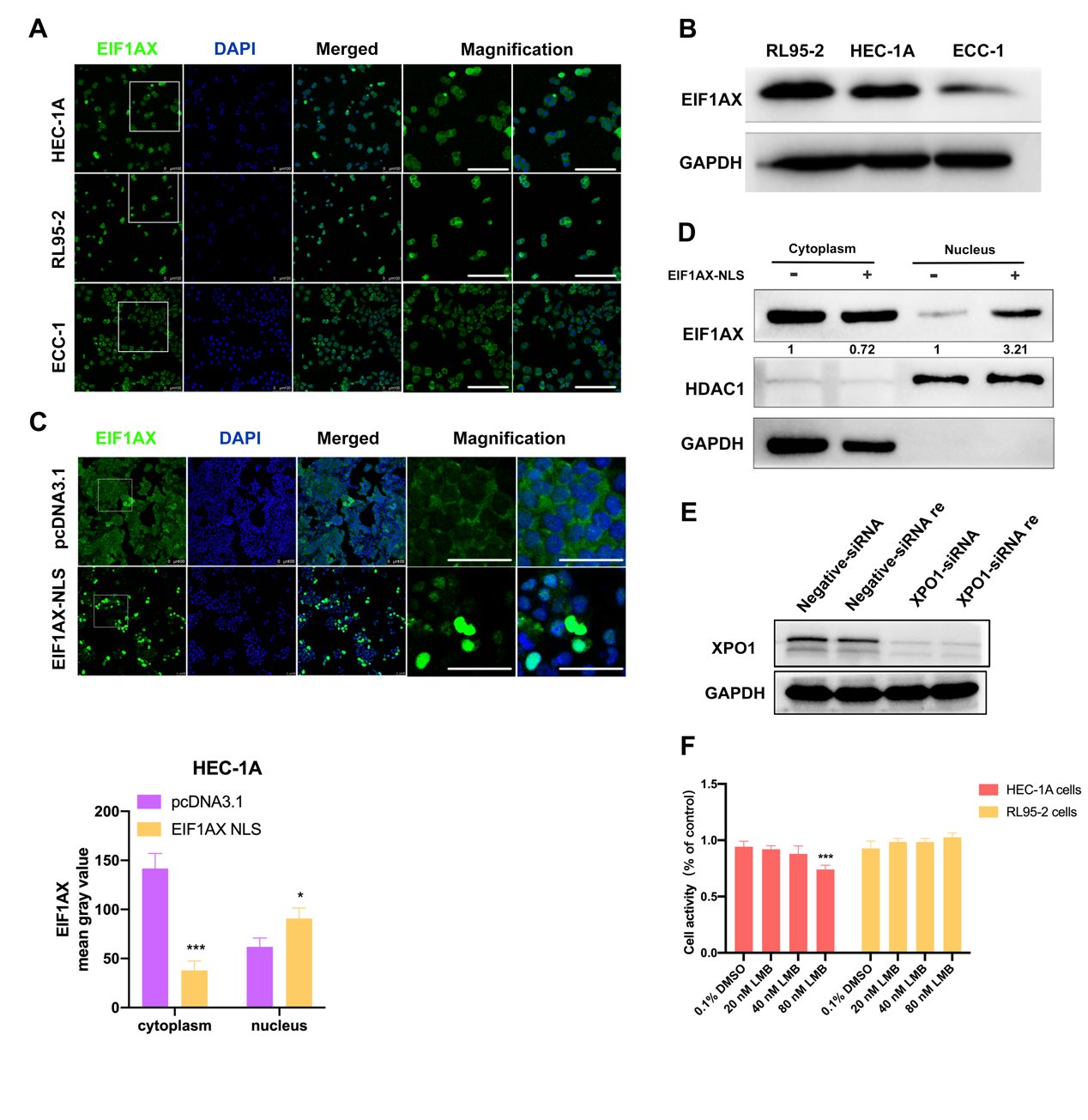
**

**Figure S4. The expression of EIF1AX or EIF1AX-SV40NLS in EC cell lines.** A: Immunofluorescence were observed the location of EIF1AX protein in HEC-1A, RL95-2 and ECC-1 cells. Scale bar :100 μm. B: Western Blot were detected the expression of EIF1AX in EC cell lines. C, D: The expression of EIF1AX protein after transfected with EIF1AX-SV40NLS plasmid. Scale bar :100 μm. Scale bar: 50 μm in magnification. Student’s *t* test: n=3, **P<*0.05, ****P<*0.001. Bars indicate SD. E: The expression of XPO1 after *XPO1* knockdown. F: CCK-8 were detected the cell activity of EC cell lines following LMB treatment for 1 h. One-way ANOVA: n=3, ****P<*0.001. Bars indicate SD.
