# Supplementary material for: XPO1-Mediated EIF1AX Cytoplasmic Relocation Promotes Tumor Migration and Invasion in Endometrial Carcinoma
Source: Oxid Med Cell Longev. 2022 Dec 22;2022:1361135. doi: 10.1155/2022/1361135 (PMC9800903; doi:10.1155/2022/1361135)
Supplement: Supplementary 2 — Supplementary Table 1: The siRNA sequences targeting IPO13 and XPO1. [file 1361135.f2.docx]

Supplementary Table 1. The siRNA sequences targeting IPO13 and XPO1

| Gene name | siRNA sequences |
| --- | --- |
| IPO13 | sense: 5’-CCAGGGAUCAUCCUGAUAUTT -3’  antisense: 5’-AUAUCAGGAUGAUCCCUGGTT-3’ |
|  | sense: 5’-CACUCGUCAACAUGAUUAUTT -3’  antisense: 5’-AUAAUCAUGUUGACGAGUGTT-3’ |
| XPO1 | sense: 5’- GCAGAUGCUUCCUUUAAAUTT -3’  antisense: 5’-AUUUAAAGGAAGCAUCUGCTT-3’ |
| Negative control | sense: 5’-UUCUCCGAACGUGUCACGUTT-3’  antisense: 5’-ACGUGACACGUUCGGAGAATT-3’ |
