# Supplementary material for: XPO1-Mediated EIF1AX Cytoplasmic Relocation Promotes Tumor Migration and Invasion in Endometrial Carcinoma
Source: Oxid Med Cell Longev. 2022 Dec 22;2022:1361135. doi: 10.1155/2022/1361135 (PMC9800903; doi:10.1155/2022/1361135)
Supplement: Supplementary 3 — Supplementary Table 2: primer sequences for amplification and sequencing of EIF1AX-CDS. [file 1361135.f3.docx]

Supplementary Table 2. Primer sequences for amplification and sequencing of EIF1AX-CDS

|  | Primer sequences |
| --- | --- |
| EIF1AX-CDS | Forward primer: 5’-AACAGACGCAGGGGTAAGAA -3’  Reverse primer: 5’-CGCCGTATGCCTTCAGACTT-3’ |
